# Supplementary material for: Identifying the NEAT1/miR-26b-5p/S100A2 axis as a regulator in Parkinson’s disease based on the ferroptosis-related genes
Source: PLoS One. 2024 Dec 31;19(12):e0316179. doi: 10.1371/journal.pone.0316179 (PMC11687868; doi:10.1371/journal.pone.0316179)
Supplement: S1 Table — (DOCX) [file pone.0316179.s001.docx]

| Gene | Forward(5’-3’) | Reverse(5’-3’) |
| --- | --- | --- |
| S100A2 | GCCAAGAGGGCGACAAGTT | AGGAAAACAGCATACTCCTGGA |
| GNGT1 | ATTACGTTGAAGAACGATCTGGC | GGATGCCCTTTACCAGTGGA |
| FCN2 | CTGCCATGTGTCAAACCTGAA | TTCCCCGACTTCCAGTTGATG |
| NEUROD4 | ACCAGGTACTTATGGGATGCT | AAGGCGAGCTTTGGTCATCTT |
| NEAT1 | GTGGCTGTTGGAGTCGGTAT | AAACCACGGTCCATGAAGCA |
| miR-7b-5p | CGCGCGGGTTGTGATTT | AGTGCAGGGTCCGAGGTATT |
| miR-26b-5p | GCGCGTTCAAGTAATTCAGG | AGTGCAGGGTCCGAGGTATT |
| GAPDH | ACAGCCTCAAGATCATCAGC | GGTCATGAGTCCTTCCACGAT |
| U6 | CTCGCTTCGGCAGCACA | AACGCTTCACGAATTTGCGT |
