# Supplementary material for: Identifying the NEAT1/miR-26b-5p/S100A2 axis as a regulator in Parkinson’s disease based on the ferroptosis-related genes
Source: PLoS One. 2024 Dec 31;19(12):e0316179. doi: 10.1371/journal.pone.0316179 (PMC11687868; doi:10.1371/journal.pone.0316179)
Supplement: S2 Table — (DOCX) [file pone.0316179.s002.docx]

| Machine Learning | Genes |
| --- | --- |
| LASSO | GNGT1, CLK2, FCN2, SGSH, NEUROD4, BRCA2, BMP3, S100A2, CDH6, RGS6, SEMA6D, DRD2 |
| RF | ABCG4, CLK2, NEUROD4, MCC, TAZ, BRCA2, FCN2, MCM5, ASB4, RAB3A, ITGAL, KCNB2, DDC, GNGT1, SDC1, CLDN15, BMP3, CHN2, RGS6, SGSH, SMPX, S100A2, RBMS2, MECR, FOXC1, VGLL3, ANK1 |
| XGBoost | EN1, GNGT1, FCN2, DRD2, RGS6, CLDN15, SGSH, CDH6, BMP3, NEUROD4, GABRA4, ST8SIA3, GFRA1, S100A2, FOXA1, FGR, PYCRL |
| GBM | GNGT1, FCN2, DRD2, EN1, BMP3, RGS6, CDH6, NEUROD4, CNTN6, SGSH, CLDN15, ACP2, S100A2, BRCA2, RAB3A, LPO, ST8SIA3, DDC, PYCRL, DGKI |
| SVM | FCN2, GNGT1, CNTN6, RAB3A, DDC, TRPC6, DRD2, TH, NEUROD4, DGKI, EN1, ASB4, SEZ6L, GABRA4, LPO, SEMA6D, GFRA1, S100A2, FOXA1, TRPV2, SDC1, RET, ABCG4, SMPX, CLDN15, ANK1, DLK1, PRMT8 |
