# Supplementary figures and images for: Identifying the NEAT1/miR-26b-5p/S100A2 axis as a regulator in Parkinson’s disease based on the ferroptosis-related genes
Source: PLoS One. 2024 Dec 31;19(12):e0316179. doi: 10.1371/journal.pone.0316179 (PMC11687868; doi:10.1371/journal.pone.0316179)

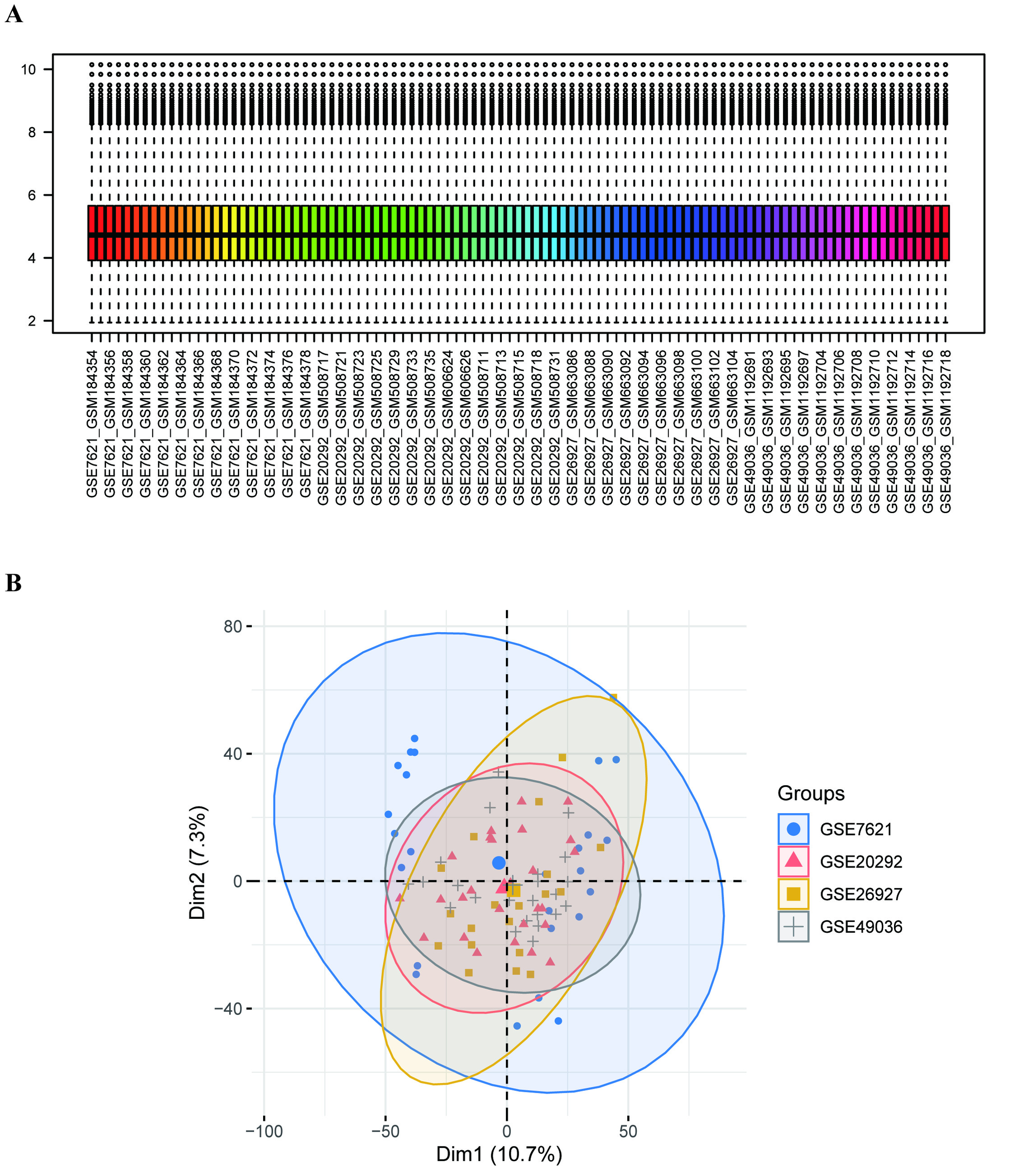

Supplement: S1 Fig — (A) Background corrected and normalized the batch effect of datasets. (B) PCA analysis of the four datasets after the normalized. (TIF) [file pone.0316179.s003.tif]

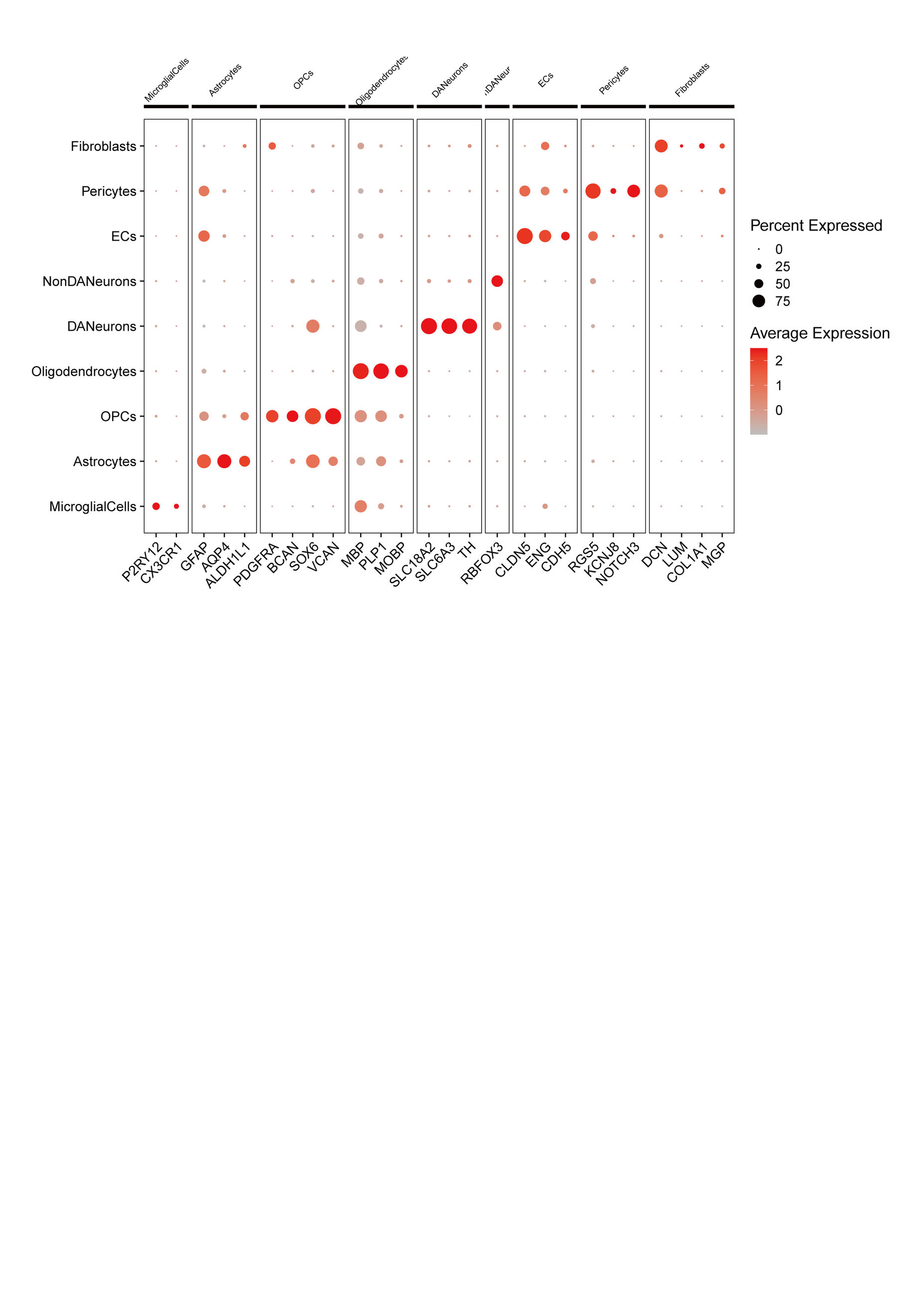

Supplement: S2 Fig — (TIF) [file pone.0316179.s004.tif]
