# Supplementary material for: Identifying the NEAT1/miR-26b-5p/S100A2 axis as a regulator in Parkinson’s disease based on the ferroptosis-related genes
Source: PLoS One. 2024 Dec 31;19(12):e0316179. doi: 10.1371/journal.pone.0316179 (PMC11687868; doi:10.1371/journal.pone.0316179)
Supplement: S1 Raw images — (PDF) [file pone.0316179.s005.pdf]

Original whole membrane of western blots in fig6 (E).
The TFR, TH, FTH1 and Actin are derived from a membrane, the entire membrane is shown below this page.

TFR

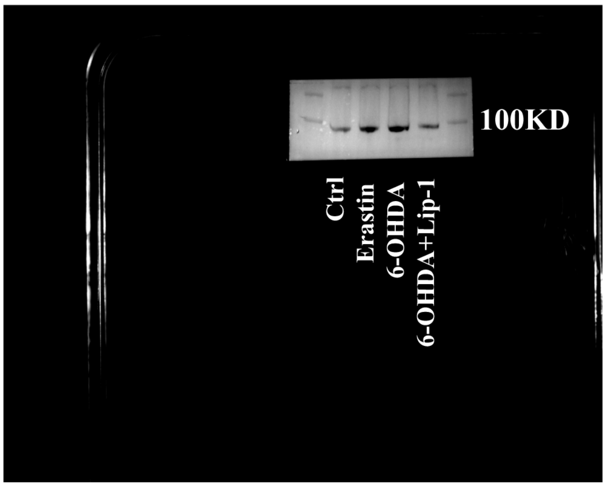

TH

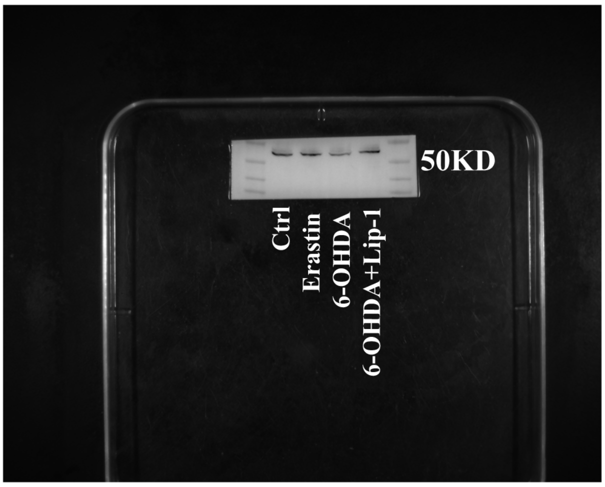

FTH1

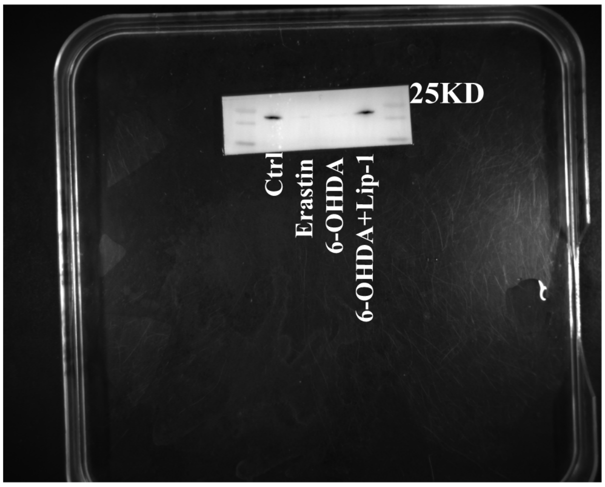

ACTIN

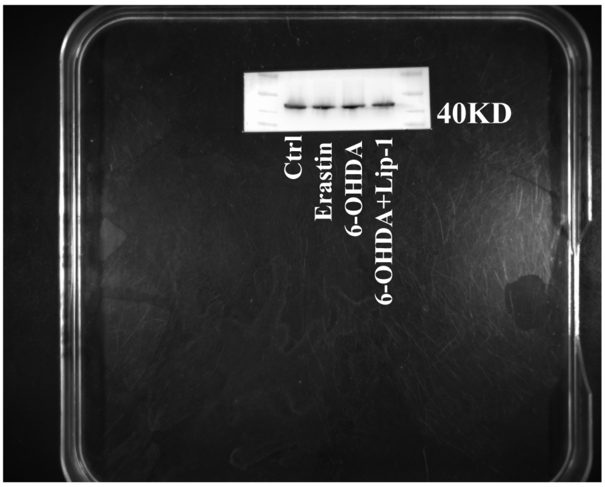

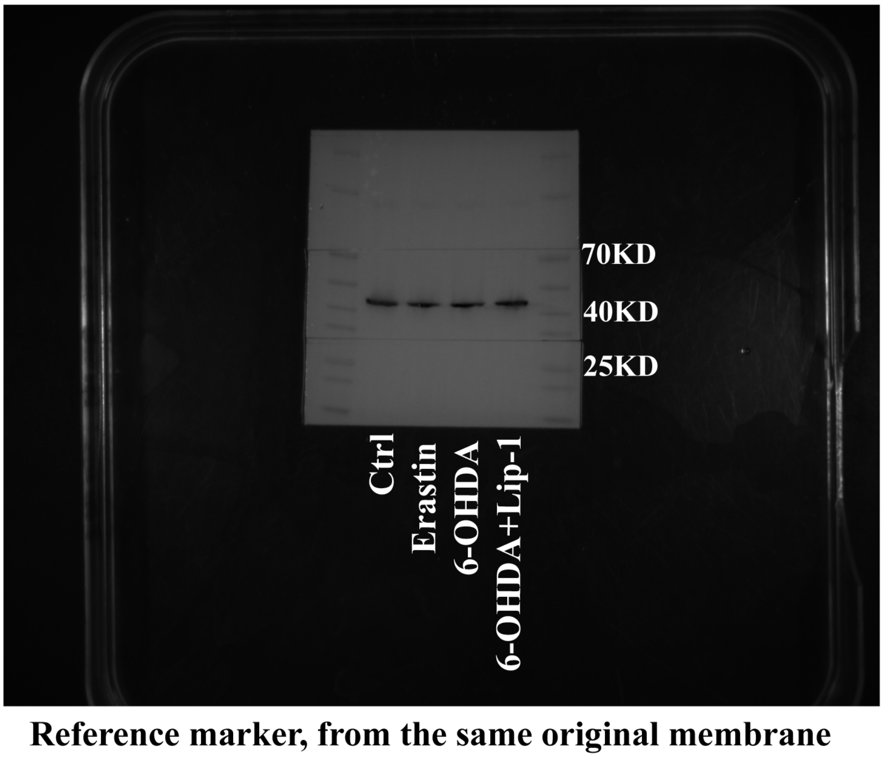

Reference marker, from the same original membrane

Original whole membrane of western blots in fig6 (E).
The ACSL4, GPX4 and Actin are derived from a membrane, the entire membrane is shown below this page.

ACSL4

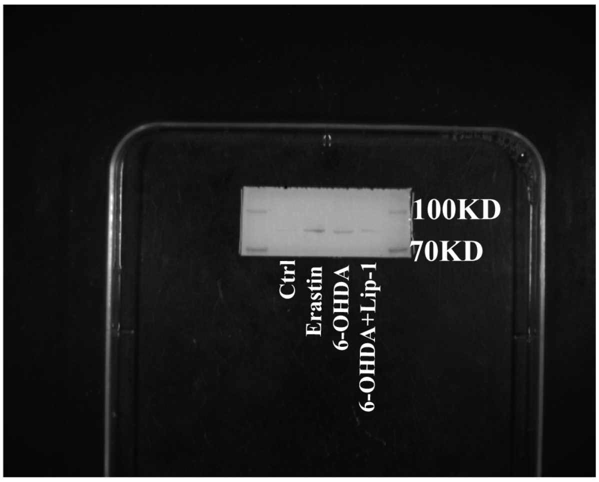

GPX4

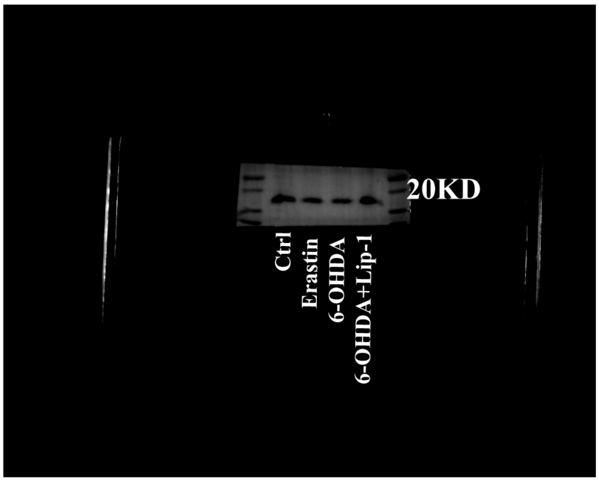

ACTIN

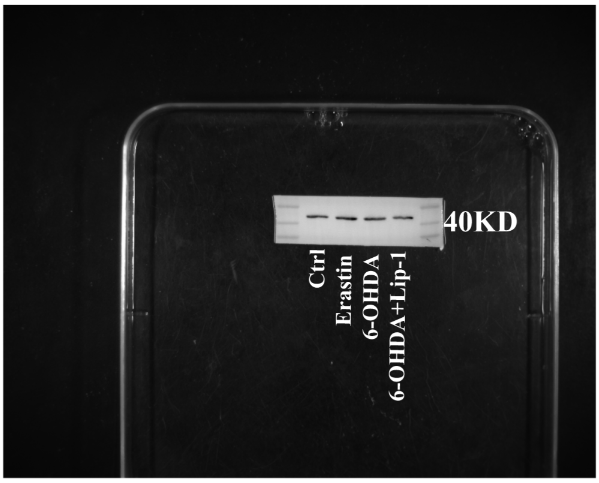

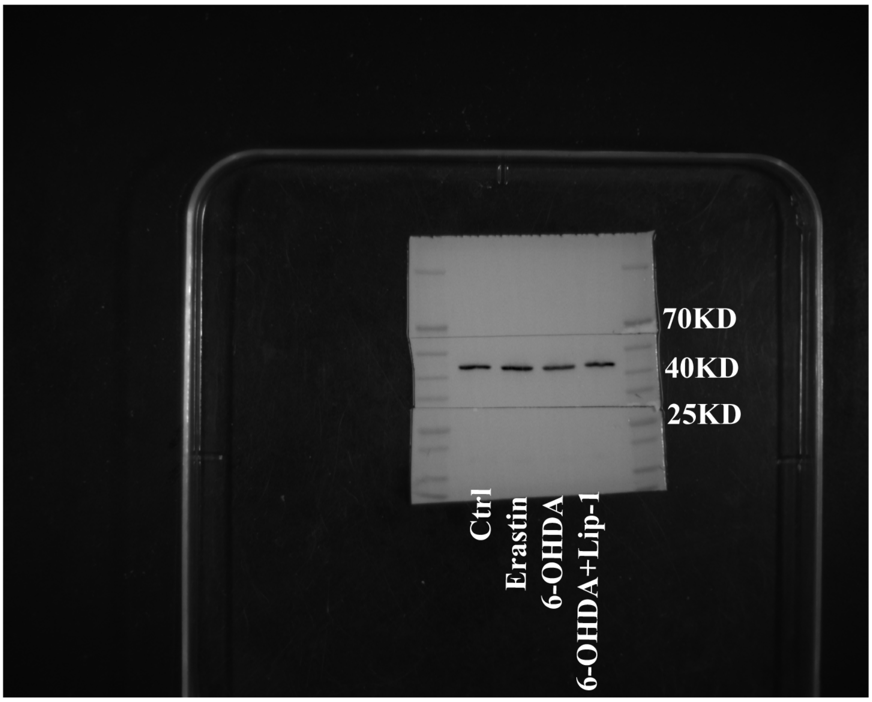

Reference marker, from the same original membrane

Original whole membrane of western blots in fig7 (I).
The left protein was derived from the same membrane as the right Actin

GPX4

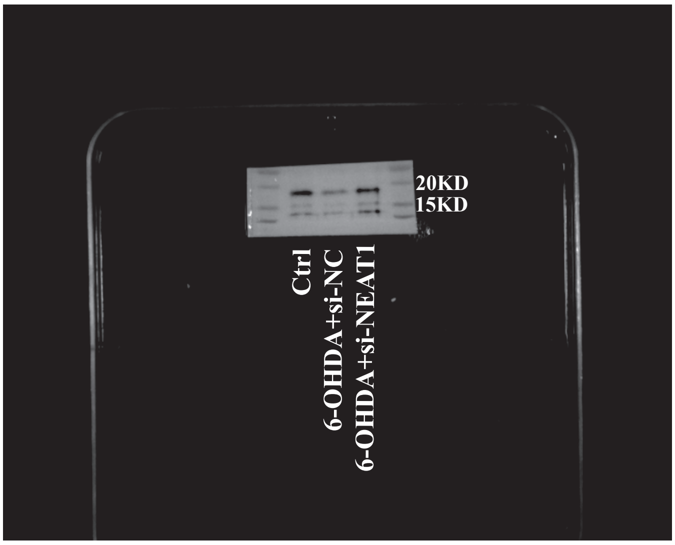

GPX4-ACTIN

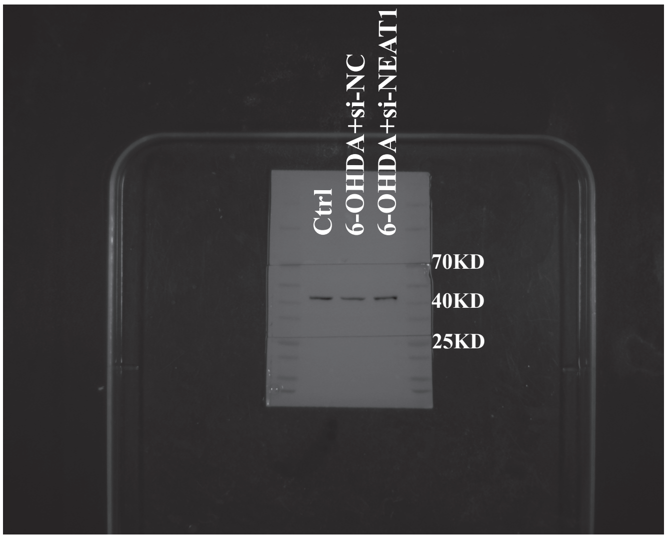

ACSL4

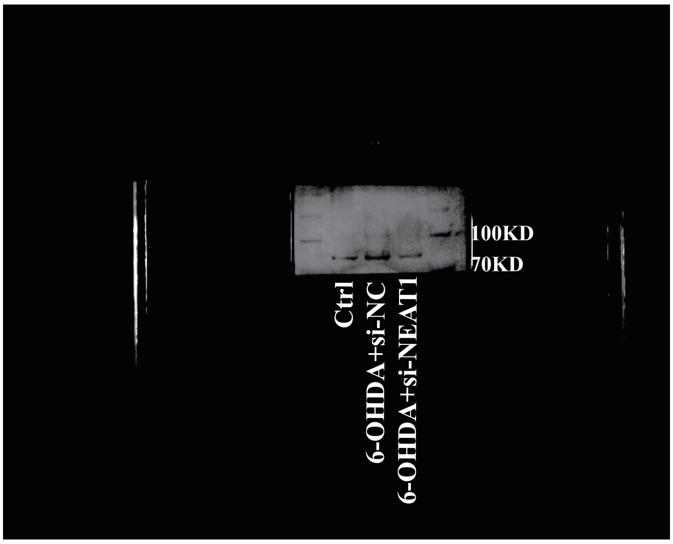

ACSL4-ACTIN

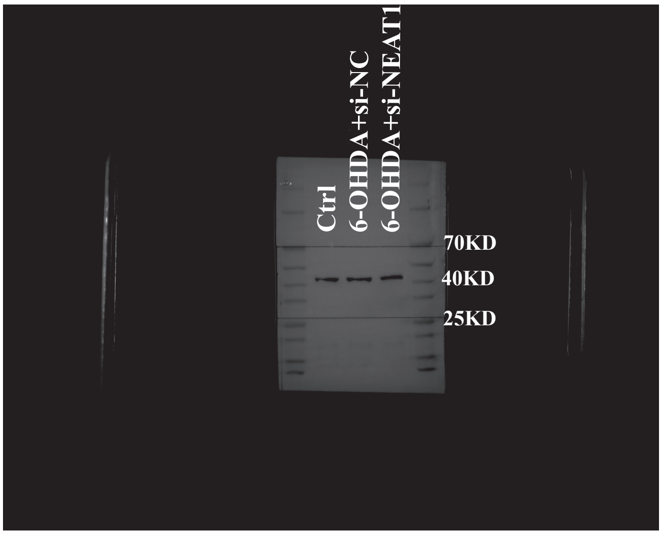

TH

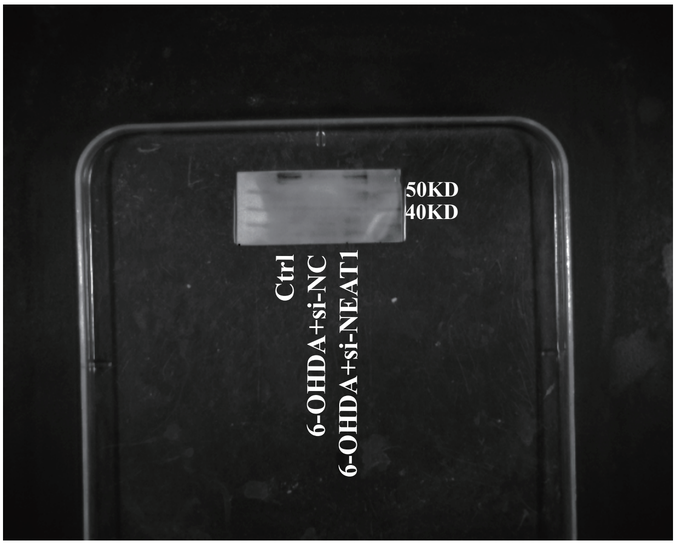

TH-ACTIN

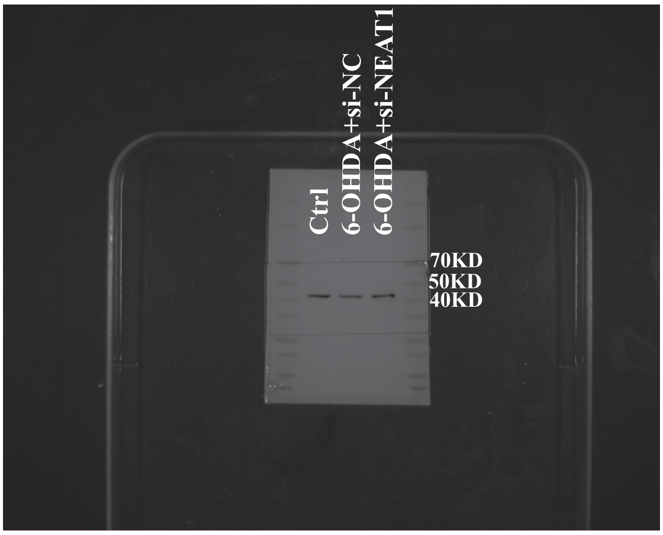

Reference marker, from the same original membrane

Original whole membrane of western blots in fig7 (I).
The TFR, FTH1 and Actin are derived from a membrane, the entire membrane is shown below this page.

TFR

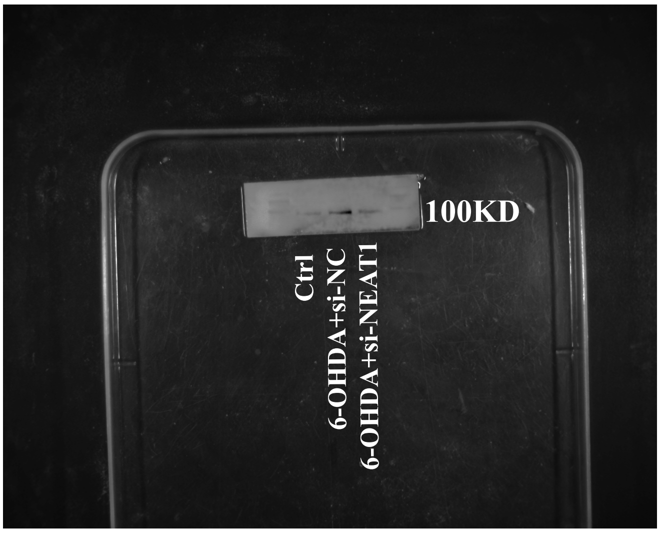

FTH1

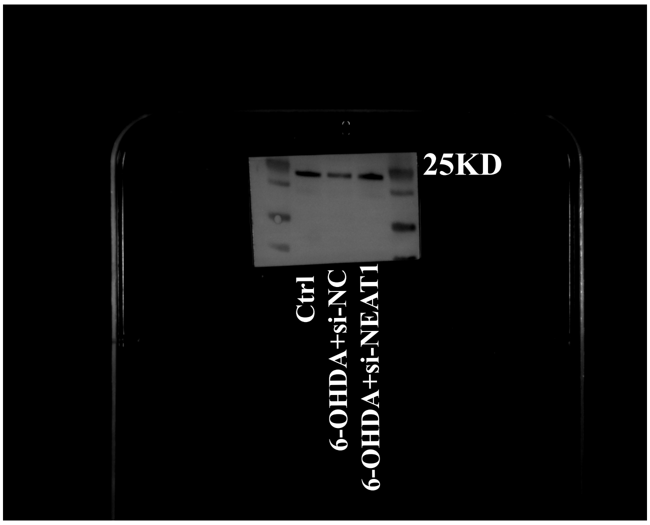

ACTIN

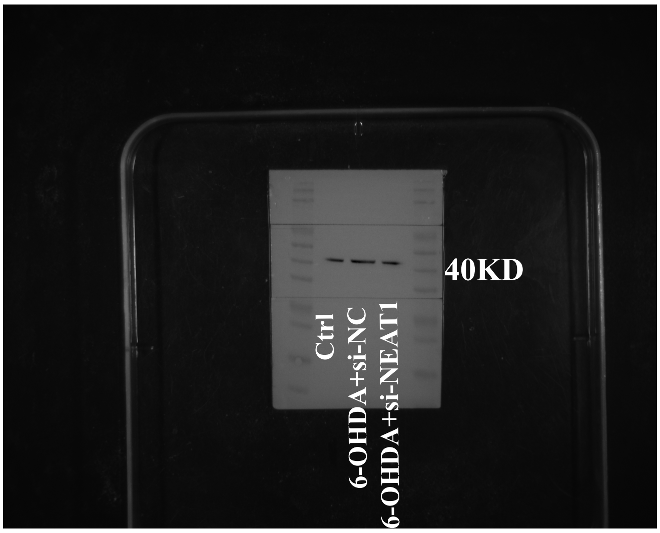

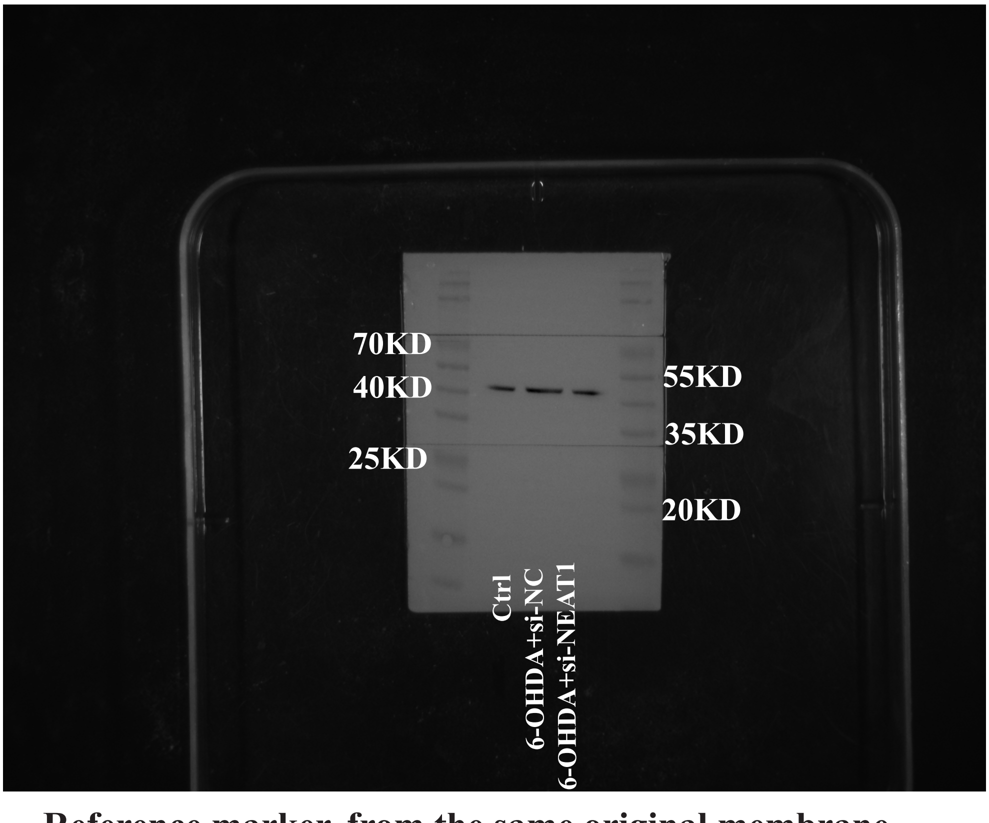

Reference marker, from the same original membrane
