# Supplementary material for: Identifying the NEAT1/miR-26b-5p/S100A2 axis as a regulator in Parkinson’s disease based on the ferroptosis-related genes
Source: PLoS One. 2024 Dec 31;19(12):e0316179. doi: 10.1371/journal.pone.0316179 (PMC11687868; doi:10.1371/journal.pone.0316179)
Supplement: Attachment — Submitted filename: Response to Reviewers.docx [file pone.0316179.s006.docx]

Dear editor’s and reviewer’s,

Thanks for your comments concerning our manuscript. They are all valuable and very important to guide our research. We have studied these comments carefully and revised the manuscript which we hope to meet with approval. Most importantly, we re-performed and added some statistical method, bioinformatic analysis especially the single-cell sequencing or experiments, which may help to fully answer the reviewer’s comments and improve the quality of the manuscript. The main revisions have been marked in the “Revised Manuscript with Track Changes”, and the “Response to Reviewers” comments are as follow. We hope our revision and the improvement of the manuscript can answer your questions.

Point-to-point response to Reviewer 1:

Question 1: The primary concern is using the sva package to remove batch effects, which is not recommended. Instead, batch should be included as a covariate in statistical models. The manuscript at https://doi.org/10.1093/biostatistics/kxv027 is highly recommended for further guidance. Programs like ComBat aim to modify the data to eliminate batch effects, but it is better to model the batch effect in the design formula without directly modifying the data.

Response: Thanks for your comment. We really appreciate your advice and it is very confirmed and valuable suggestion. It is better to include batch effects as a covariate in all statistical tests instead of removing batch effects directly with “ComBat” or “removeBatchEffect” etc. The combat modifies the expression of the entire matrix through batch effects, which sometimes may lead to negative values or overfitting. We apologize for the lack of clarity in the methods section and we modified it. Initially, limma allows batch effects, like any other factor, to be included as covariates during normalization (providing the batch information in the limma and DESeq2 packet grouping information) were performed, however, the effect is not good. Thus, we attempted to optimize the “ComBat” algorithm of the sva package. During the data preprocessing, we firstly performed background corrected on each dataset, the quality normalized were achieved after merging them. We used the model.matrix() function to construct a design model that should include the batch information as the covariates, and the Bayesian approach were used to estimate the batch effects (Setting par.prior=TRUE). We also performed Combat multiple times then choose the best result. We have added the results of the batch effect removal in supplementary figures and the boxplot indicates that the batch effect has been significantly eliminated.

Question 2: The methodology section lacks clarity and detail in certain areas. For instance, the specific methods used to correct the background and normalize the data are unclear. It is also uncertain whether any filters were applied to exclude probes with no expression or if quality control measures such as principal component analysis were conducted.

Response: Thanks for your comment and we sincerely apologize for the lack of clarity in the methodology section. We have made detailed revisions to the 2.1 section (Data Collection and Processing). We elaborated the corresponding R packages and algorithms that used for the background correction, probes filter, datasets transformation or normalization and others in detail.

We also included the diagram of the principal component analysis in the supplementary figures.

Question 3: the authors do not appear to have addressed potential confounding factors, and it seems that multiple testing corrections were not utilized. The rationale for selecting |log2 fold change | > 0.1 as the threshold for differential expression analysis is unclear.

Response: Thanks for your suggestion and it is a very correct and valuable suggestion. We sincerely apologize for the lack of clarity in our explanation of the DEGs analysis methods and for the limitations present in it. We used the R package “limma” for DEGs analysis. We constructed the expression matrix based on the confounding factors, used the “lmfit” function to fit a linear model, and conducted Bayesian testing. The p-value was adjusted using the BH method in order to control the FDR. However, the correction results are not very satisfactory, and the number of DEGs (adjust p<0.05) is relatively small. The reason may be related to the different batch sources of our samples after correction and the small sample size. Furthermore, in neurodegenerative diseases such as parkinson's disease, subtle changes of DEGs may lead to alterations in many related pathological pathways and molecular biology mechanisms. Therefore, we set a broader threshold for logFC or p-values, and we validated the hub genes through the experiment. We apologize for the confusion this has caused to you, we have revised the methodology section and discussed it in the limitations.

Question 4: The authors should also clarify the reasoning behind the selection of datasets, as the WGCNA analysis was carried out using only one dataset, while the DEGs analysis involved different datasets.

Response: Thanks for the invaluable comment and this is a detail worth clarifying. WGCNA was used to evaluate the relationships between hub modules and clinical traits of PD. The quality requirements for the samples are high. The four datasets are derived from sequencing conducted by different platforms or companies. We attempted to perform WGCNA on the different datasets, however, due to the large sample size and number of genes, the resulting gene modules seems that were not very satisfactory. Compared to other datasets, The GSE20292 has a better quality and the sample size meets the requirements for WGCNA, particularly with well-documented clinical information. Furthermore, there are no outlier samples or anomalous values, and the patients and control groups are matched in terms of sex and age. Therefore, we believe that conducting WGCNA on this dataset can yield modules significantly related to clinical features of PD. Thanks for your suggestions, and we have also made corresponding revisions and explanations to the 2.5 section.

Question 5: The authors are strongly encouraged to review the "Minimum Information for Publication of Quantitative Real-Time PCR Experiments" (MIQE) guidelines. The MIQE publication provides detailed information on the minimal and desirable reporting requirements for qPCR data publication.

Response: Thanks for your suggestions and they are extremely valuable. We have studied and referenced the MIQE guideline and related literatures. We have rewritten the quantitative real-time PCR methods and the details refer to the 2.13 section.

Point-to-point response to Reviewer 2:

Question 1: The manuscript would benefit from including single-cell expression analysis of NEAT1, NEUROD4, GNGT1, FCN2, and S100A2 in single-cell human post-mortem tissue (Kamath et al., Nature Neuroscience, 2022) and in single-cell midbrain organoids (Fiorenzano et al., Nature Communications, 2021). This addition would not only strengthen the authors’ findings but also highlight the potential use of brain organoids in future studies to explore the identified regulatory axis in more physiologically relevant conditions.

Response: We sincerely thanks for your suggestions and they are extremely valuable and it is very helpful for our research. Kamath et al developed a protocol to enrich and transcriptionally profile DA neurons from patients with PD and matched controls, sampling a total of 387,483 nuclei, including 22,048 DA neuron profiles (Kamath et al., Nature Neuroscience, 2022). Fiorenzano et al focused to a greater extent on the developmental processes of dopaminergic neurons (Fiorenzano et al., Nature Communications, 2021). Based on the database GSE178265 (ncbi.nlm.nih. gov/geo/query/acc.cgi?acc=GSE178265) provided by Kamath et al, we conducted further validation of the feature genes through single-cell sequencing analysis. We have added the relevant methods, results and discussion sections. We confirmed that NEAT1 is significantly upregulated in dopaminergic neurons in PD and further clarified its distribution within cellular subpopulations, which providing new directions for exploring the molecular biological mechanisms of PD. Additionally, we found that the distribution differences of some proteins within the dopaminergic neuron subpopulations are not very pronounced. Since our another study is conducting an in-depth exploration of the molecular mechanisms of one of the feature genes, which involves aspects of single-cell sequencing. We sincerely apologize for only supplementing the relevant results regarding NEAT1 here. Thank you very much for your suggestions, and we hope that our future research can also benefit from your guidance.

Question 2: The Materials and Methods section, particularly regarding the experimental use of 6-OHDA, requires further detail and clarification to improve reproducibility and transparency.

Response: Thank you for your comments and they are very crucial for our research. We sincerely apologize for the omissions in our description. We have revised the materials and methods related to 6-OHDA administration and added relevant details.

Question 3: The use of SH-SY5Y cells may not fully capture the complexity of the identified axis. It would be beneficial for the authors to show tyrosine hydroxylase (TH) expression and potential fragmentation at the immunofluorescence level before and after 6-OHDA treatment. This would provide a more comprehensive evaluation of the cellular model and the relevance of the regulatory pathway under study.

Response: We sincerely appreciate your suggestion. it is very valuable and will be useful as we further explore the molecular biology mechanisms. The reduction of TH in dopaminergic neurons is a significant feature of PD. Previous studies have demonstrated that the 6-OHDA, serves as an specific inducer for PD models, could significantly inhibit the TH expression at the immunofluorescence levels (He X, et.al.Front Pharmacol. 2021), (Guo S, et.al.Med Princ Pract. 2024). We also validated that 6-OHDA can inhibit the expression level of TH in SH-SY5Y cells through the western blot experiments. We found that compared with the control or erastin group, expression of TH was decreased in the 6-OHDA group. It indicated that the PD cell model has been successfully established under the 6-OHDA induction, which can be used for further exploration of the molecular biological mechanisms. Thank you sincerely for your reminding and attention.

Point-to-point response to Reviewer 3:

Question 1: The machine learning model validation process needs more detail. While you mention using training, test, and external validation sets, the manuscript lacks information on cross-validation procedures, which are crucial for ensuring model robustness.

Response: Thanks for your comments and the suggestion is extremely valuable and critical. We apologize for the confusion caused by our nonstandard description of machine learning. We have modified the material and methodology section related to machine learning, added to the cross-validation procedures and relevant parameters or criteria for machine learning. The details refer to the 2.7 section.

Question 2: The biological significance of the identified NEAT1/ miR-7b-5p/S100A2 axis in PD were verified in SH-SY5Y cell line. However, it's important to acknowledge the limitations inherent in all in vitro models when interpreting these results in the context of human PD. Additional experimental validation in more convincing models (in vivo PD model, iPSC-derived model or human sample if possible) would strengthen your conclusions. Otherwise, please address the limitation of your study clearly in the manuscript.

Response: Thanks for your comments and your suggestions are very valuable and helpful for our follow-up researches. We supplemented some single-cell sequencing results to support our conclusions but we apologize that in vivo and iPSC-derived model experimental verification was not performed in this study. We have explained this in the limitations section. We will further explore the molecular biological mechanism in vivo model in the future, and we also looking forward to your guidance in our future research.

Question 3: Some of the figures contain information that is unclear or illegible. Please ensure that all submitted figures meet the required resolution standards, and provide high-resolution original images for each figure.

Response: Thanks for your comments and we are very sorry for the inconvenience caused by the quality problems of the images. We have checked and uploaded higher resolution images.

Question 4: Please provide a more comprehensive discussion of the study's limitations.

Response: Thanks for your comments and they are extremely valuable. We have added the discussion of the limitations in the last paragraph.

Question 5: The use of ROC curves and AUC for diagnostic model evaluation is appropriate. The typical way to assess a diagnostic method with ROC is to calculate the AUC for interpretation and select the optimal threshold to balance the sensitivity and specificity. Your manuscript lacks this detailed analysis. Additionally, you describe the ROC as showing "feasibility and capability" rather than the standard "sensitivity and specificity". Please revise this to use standard terminology, or provide a clear rationale for the alternative phrasing.

Response: Thanks for your suggestions and we sincerely apologize for the confusion caused by our non-standard writing. The suggestion is extremely valuable for our research. We have made corresponding revisions to the methods and results sections related to ROC, especially in terms of wording and the description of additional details.

Question 6: There's no discussion of potential overfitting or how it was addressed. Given the relatively small sample size, this is an important consideration.

Response: Thanks for your valid suggestion that is very valuable and crucial for our research. The sample size of this study is relatively small, so we used 10-fold or 5-fold cross-validation and regularization techniques to limit the model weights in machine learning to reduce overfitting. We have provided additional explanations in the methodology section related to machine learning.

Question 7: You state that all data are fully available without restriction, which is commendable. However, please ensure that all data, including any code used for the bioinformatics analysis, are made available in a public repository with clear access instructions in the manuscript.

Response: Thanks for your comments and that is very important suggestion. We have appended a description that provide the public website of all the databases, genesets or the associated codes. The details are described in the statement of the data availability section. Thank you sincerely for your reminding and attention.

Question 8: There are occasional grammatical errors and awkward phrasings.

Response: We sincerely appreciate your suggestions and apologize for the confusion caused by the grammatical errors in our writing. We have reviewed the entire manuscript and made revisions to the wording and polished the relevant statements.

Question 9: Some sentences are long and complex, which can make them difficult to follow. For example, in the abstract:"We aimed to identify the ferroptosis-related genes (FRGs) associated with PD and construct competing endogenous RNA (ceRNA) networks by using bioinformatics analysis to further assess the pathogenesis of PD." Breaking these into shorter, more focused sentences could improve readability.

Response: Thank you for your suggestions and they are very valuable. We have revised and simplified some lengthy sentences in the the abstract and main text.

Point-to-point response to Reviewer 4:

Question 1: The first author, Dr. Li is also the second author of a similar paper titled "Identifying the potential genes in alpha synuclein driving ferroptosis of Parkinson’s disease" published last year. It is not so surprising that the conclusion is different between the past paper and the current manuscript since a distinct number of databases were used. The fact clearly indicates that the conclusion is dependent on the databases. This point should be stated in the discussion section in addition to the citation of the past paper.

Response: Thanks for your comments and the suggestion is extremely valuable. The article is the foundational work of our preliminary research and only uses one dataset. In this article, we incorporated more datasets for our research and reached conclusions. It is evident that different datasets may select genes that differ. We have elaborated this in the discussion and limitation sections and we appreciate your guidance.

Question 2: The authors found that both miRNA, miR-7b-5p, and miR-26b-5p have a close relationship with the onset of Parkinson's disease. The explanation of these miRNAs seems to be strange in that miR-7b-5p appears in the title but only miR-26b-5p was discussed in the final section. Please put these matters in good order.

Response: Thanks for your comments and we sincerely apologize for the confusion caused by our writing error. We have concluded that NEAT1/miR-26b-5p/S100A2 axis is associated with ferroptosis in the pathogenesis of PD. The miR-26b-5p, not miR-7b-5p mainly plays a regulatory role. We have corrected two writing errors in the title and abstract and we sincerely apologize again for any inconvenience caused.

Question 3: The authors proposed NEAT1/miR-26b-5p or miR-7b-5p/S100A2 axis as the biomarkers but it can not be applicable in a clinical setting, because these expression profiles are only available in autopsy cases. The description in the final paragraph should be changed into a more rational one.

Response: Thanks for your comments and your suggestion is very accurate and valuable. The results obtained from the autopsy have certain limitations in clinical applications. We have revised the final paragraph and elaborated on it in the limitation supplementary part.

Question 4: Multiple machine-learning methods were employed to select the candidate genes. It is not necessarily obvious how the authors selected 12 genes in Figure 5C/D, 30 genes in Figure 5E, 28 indicators in Figure 5F, 36 variables in Figure 5G, and 20 genes in Figure 5H. In Figure F, the elbow can be seen around the No. 8 or 9 variable. It is reasonable to show the criteria.

Response: Thanks for your comments and this is a very valuable and critical suggestion. We sincerely apologize for the confusion caused by our lack of proper writing. We have re-conducted the machine learning and identified some oversights, however, these do not affect our results. We have revised the methodology and results sections related to machine learning. We have added the relevant parameters or criteria for machine learning, and the details refer to the 2.7 and 3.6 section.

Question 5: In Figures 5G and 5H, the names of the selected genes are hardly visible. The gene names "S100A2, GNGT1, NEUROD4, and FCN2" can only be seen in Figure 5E. Please show the names in 5G and 5H visibly if possible.

Response: Thanks for your comments and we sincerely apologize for the inconvenience caused. We have optimized and uploaded the highest resolution images. Furthermore, We have provided a table list of genes obtained from the five machine learning algorithms in 3.6 section.

Question 6: There is a statement "The violin plot indicated that the GNGT1, FCN2, and NEUROD4 were expressed at low levels, while S100A2 was expressed at high levels in PD patients (Figure 6A-D)." However, the reviewer could not find the corresponding "violin" plot.

Response: Thanks for your comments and we sincerely apologize for the inconvenience caused by our writing error. We used a bar chart to show the expression levels of feature genes and we mistakenly wrote "violin plot" instead of "bar chart". We have made the modifications to the relevant statement.

Question 7: There is a typo of "alzheimer's" on page 18 in line 11. "a" should be written in the capital letter "A".

Response: We sincerely appreciate your careful review of our manuscript, and we sincerely apologize for any inconvenience caused by our spelling or writing errors. We have made the necessary corrections.
